# The prevalence of mental illness in young people in custody over time: a comparison of three surveys in New South Wales

**DOI:** 10.1080/13218719.2023.2192257

**Published:** 2023-05-31

**Authors:** Carey Marr, Claire Gaskin, John Kasinathan, Sharlene Kaye, Yolisha Singh, Kimberlie Dean

**Affiliations:** a School of Clinical Medicine, Discipline of Psychiatry and Mental Health, University of New South Wales, Randwick, NSW, Australia; b Justice Health and Forensic Mental Health Network, University of New South Wales, Randwick, NSW, Australia; c National Drug and Alcohol Research Centre, University of New South Wales, Randwick, NSW, Australia

**Keywords:** adolescent mental illness, adolescent self-harm, adolescent suicide, childhood trauma, custodial settings, prevalence rates, substance use, trends over time, youth justice

## Abstract

Few studies have examined the prevalence of mental illness in young people over time within the same jurisdiction. In the current study, we compared data from three large surveys of youth in custody in New South Wales, conducted in 2003, 2009 and 2015. We examined rates of mental illness, self-harm and suicidal behaviours, substance use and childhood trauma and found little consistent change over time, though some fluctuations were observed regarding certain mental illnesses and substance use. We also descriptively compared findings with observed rates for the general population and found that young people in custody showed higher levels of all examined variables. In sum, these data suggest little improvement in the well-being of young people in custody in New South Wales over time. Better identification and treatment of these issues are vital if young people are to be prevented from becoming enmeshed in the criminal justice system.

## Introduction

Across the world, mental illness is known to be highly prevalent in youth detention and custodial settings. One recent meta-analysis examined 47 studies of adolescents in detention and found elevated rates of several disorders (Beaudry, Yu, Långström, & Fazel, 2021). This included disorders such as psychotic illness (3% in incarcerated males and females vs. 0.5–1.5% in general population), major depressive disorder (MDD; 9% in incarcerated males and 27% in incarcerated females vs. 10% and 26% in general population), attention deficit hyperactivity disorder (ADHD; 17% incarcerated males and 20% incarcerated females vs. 11% in general population), post-traumatic stress disorder (PTSD; 8% in incarcerated males, 17% in incarcerated females vs. 2% and 8% in general population), and lifetime conduct disorder (62% in incarcerated males and 63% in incarcerated females vs. 10% in general population). In addition, self-harm and suicidal behaviours were found to be elevated in detained adolescent samples, with higher rates of suicidal ideation (19.0–38.3% vs. 15.3% in general population), attempts (17.3–39.8% vs. 4.1% in general population), suicide deaths (17.6–32.0% vs. 6.0–7.8% in general population) and increased rates of self-harm reported (20.9–47.1% vs. 10.5–16.9% in general population; Borschmann et al., 2020).

Individual studies conducted in Australia have similarly found high rates of mental illness and self-harm and suicidal behaviours in young people in custody. For example, a study of 80 adolescent boys and 20 adolescent girls in custody (aged 12–20 years) found that 86% had at least one diagnosis as defined by the *Diagnostic and Statistical Manual of Mental Disorders–Third Edition, Revised* (DSM–III–R; APA, 1987) criteria (Richards, 1996). Another study using the Adolescent Psychopathology Scale found similar results: young people in custody in Tasmania (aged 12–18 years) had rates of mental illness more than five times those of adolescents in the community (Bickel & Campbell, 2002). High rates of mental illness in incarcerated young people were also found in Australian studies where other clinical scales were used, including the Kiddie Schedule for Affective Disorders and Schizophrenia for School-Age Children–Present and Lifetime version (K–SADS–PL; Dixon, Howie, & Starling, 2004), the Massachusetts Youth Screening Instrument (Version 2; MAY–SI–2; Stathis et al., 2008) and the Structured Clinical Interview for DSM–IV–Screen (SCID–Screen; Fougere, Thomas, & Daffern, 2013). Another large study examined such behaviours in 900 young people admitted to South Australia’s two detention centres (aged 11–20 years; Putnins, 2005). Over a quarter of the sample reported recent suicidal ideation (26%) and having attempted suicide in the past (27%), showing increased levels of self-harm and suicidal behaviours in young people in custody when compared with young people in the general population.

The recent systematic review and meta-analysis conducted by Beaudry et al. (2021) served as an update to a previous review of this topic by Fazel, Doll, and Långström (2008). A comparison of these reviews shows fairly stable prevalences of mental illness over time in youth detention settings worldwide (Beaudry et al., 2021). However, few studies have examined the prevalence of mental illness in young people over time within the same jurisdiction (but see Browne et al., 2022, for such an analysis of adults in custody in New South Wales, NSW). One past study examining young people on remand in South Australia found little evidence of change in prevalence of mental health problems over a 20-year period (Sawyer et al., 2010). General population studies of young people in Australia have suggested no changes or slight increases in the prevalence of mental illness over time (e.g. Lawrence et al., 2015; Mission Australia, 2015, 2017). Still, no studies have yet examined trends in the prevalence of mental illness in samples of young people in custody over time in Australia. Knowledge of rates over time within one system is valuable not only for indicating trends in rates of mental illness in incarcerated young people, but also for improving service planning and intervention.

## The current study

In this study, data from three large surveys of youth in custody in New South Wales (NSW) conducted in 2003 (*2003 NSW Young People in Custody Health Survey: Key Findings Report*, 2003), 2009 (Indig et al., 2011) and 2015 (*2015 Young People in Custody Health Survey: Full Report*, 2017) are compared in terms of rates of mental illness, self-harm and suicidal behaviours, and other relevant factors. Comparisons are also made to published time trends for young people in the general population. The current study was an exploratory study, and previous surveys were reported in a stand-alone fashion, thus a priori hypotheses regarding trends over time were not possible.

## Method

### Sample

In this study, the NSW Young People in Custody Health Survey (YPICHS) samples across three consecutive surveys (conducted in 2003, 2009 and 2015) were compared. These surveys were conducted by the Justice Health and Forensic Mental Health Network as part of a strategy to inform health service developments for young people in custody. Though individual reports were created upon completion of each survey, the current study is the first to comprehensively examine changes over time across the three surveys, allowing a broader scope for future service planning.

In each survey, a total population sampling approach was taken, such that all young people remanded or sentenced to custody across New South Wales during the three-month period of the surveys were considered potential participants. The number of Juvenile Justice Centres/Juvenile Correctional Centres differed over the years due to restructuring. In 2003 and 2009, participants were recruited from nine centres, and in 2015, participants were recruited from seven centres. Today, there are six operating (now called) Youth Justice Centres in NSW.

Inclusion criteria were broadly consistent across surveys. Participants were excluded in circumstances where individuals were unable to be safely interviewed or were likely to lack capacity to consent to participation (i.e. due to the presence of severe mental health problems, acute substance withdrawal, severe intellectual impairment or being considered too violent or disruptive by custody centre staff). English proficiency sufficient to enable participation in the survey was also required for inclusion. Despite potential eligibility for inclusion, a proportion of young people were unable to be approached by research staff due to competing commitments (e.g. court appearances, legal or other appointments, work commitments) or because they were released from custody on the day of the survey.

Data were collected from both male and female young people in custody between January and March in 2003, August and October in 2009, and September and December in 2015. Due to the small number of females that participated in the 2003 survey, in 2009 the data collection phase was extended for the one female centre beyond the period set for recruitment at the male centres. Young people who participated received 10 Australian dollars in compensation for their time.

In 2003, 319 young people were eligible for inclusion, and 75.9% participated, leaving a final sample of 242 (223 males, 19 females). In 2009, 382 of 452 young people in custody were invited to participate in the survey, and 86.6% consented, leaving a final sample of 331 (290 males, 41 females). In 2015, 251 of 383 young people in custody were invited, and 90.4% consented, leaving a final sample of 227 (207 males, 19 females, 1 unknown). Thus, the total sample across all three surveys was 800 (720 males, 79 females, 1 unknown).

### Measures

**Figure 1.**
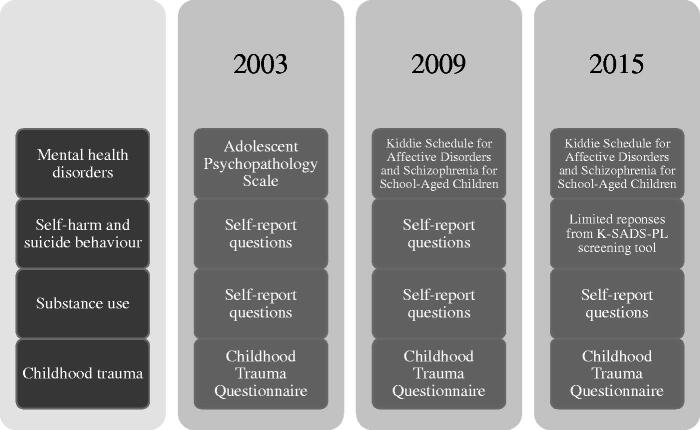
Measures undertaken across survey years.

The YPICHS collected a variety of data related to the health of young people in custody in NSW, including physical examinations, dental examinations (in 2003 and 2009; assessed via self-report in 2015), physical health, mental health and cognitive assessments, testing of blood and urine specimens and questionnaires relevant to offending behaviour and demographics. Participants were able to opt out of any of these study elements or skip any questions they did not wish to answer. Interviews and assessments were conducted face-to-face. In this study, our analysis focused on data relating to participants’ mental health, self-reported self-harm and suicidal behaviour, drug and alcohol use, and traumatic childhood experiences. Figure 1 presents the measures used for each category within each survey.

We also examined key demographic variables, including gender, Aboriginal and/or Torres Strait Islander background, age, country of birth, language spoken at home, school attendance prior to incarceration, history of parental incarceration and whether the young person had ever been placed in care. Additionally, we assessed criminal justice variables, including the young person’s legal status at the time of interview, whether they had previously been in custody and whether their most serious charge for the current period of incarceration was for a violent or non-violent offence.

#### Mental health disorders

In 2003, the Adolescent Psychopathology Scale (APS; Reynolds, 1998) was used to examine current mental illness. Although not a formal diagnostic tool, the APS is based on *Diagnostic and Statistical Manual of Mental Disorders–Fourth Edition* (DSM–IV) criteria, and clinical scores were calculated ranging from no symptoms to severe symptoms. In our analysis, we focused on common Axis I disorders, which included disruptive behavioural disorders (i.e. attention deficit hyperactivity disorder (ADHD); conduct disorder; oppositional defiant disorder (ODD); anxiety disorders (i.e. panic disorder, obsessive-compulsive disorder (OCD); generalised anxiety disorder (GAD); social phobia; separation anxiety; post-traumatic disorder (PTSD); mood disorders (i.e. major depressive disorder (MDD); dysthymic disorder; mania); psychotic disorders (i.e. schizophrenia) and substance use disorders (i.e. alcohol and/or other substance misuse). We used scores of moderate to severe to indicate likely mental illness (in line with Robertson, Dill, Husain, & Undesser, 2004).

In 2009 and 2015, more formal health diagnoses were obtained using the Kiddie Schedule for Affective Disorders and Schizophrenia for School-Age Children–Present and Lifetime version (K–SADS–PL; Kaufman et al., 1997). The K–SADS–PL is a semi-structured diagnostic interview that identifies disorders based on DSM–IV criteria. The lifetime (i.e. past diagnoses) portion was only conducted in 2009; thus, our analysis focused only on assessment of current psychopathology (i.e. within the past 12 months). Trained psychologists and mental health nurses with experience in custodial settings administered the K–SADS. The parent and teacher sections of the K–SADS were not administered. Included disorders were classified into the same five groups, including disruptive behavioural disorders (i.e. ADHD, ADHD not otherwise specified, conduct disorder, ODD, disruptive disorders not otherwise specified), anxiety disorders (i.e. panic disorder, OCD, GAD, agoraphobia, simple phobia, social phobia, separation anxiety, PTSD, acute stress disorder, anxiety disorders not otherwise specified), mood disorders [i.e. major depression, dysthymic disorder, other depressive disorder, mania, mixed episodes (specifically, major depression and mania or mania and hypomania), bipolar I, bipolar II, bipolar not otherwise specified and cyclothymia], psychotic disorders (i.e. schizophrenia, schizoaffective disorder, schizophreniform disorder, psychotic disorders not otherwise specified) and substance use disorders (i.e. alcohol abuse, alcohol dependence, substance abuse and substance dependence).

#### Self-harm and suicidal behaviour

In the 2003 and 2009 interviews, participants were asked several questions about self-harm and suicidal behaviours. In this study, we assessed the self-report responses regarding lifetime history and recent experiences (i.e. within the past 12 months) of self-harm or suicidal ideation or attempts.

The self-report questions asked in 2015 focused only on behaviours occurring since the young person most recently entered custody and were therefore not suitable for comparison with previous years. However, participants in the 2015 survey took part in a self-harm and suicidal behaviour screening that was part of the K–SADS–PL. Thus, though not directly comparable with the self-reports from 2003 and 2009, we reported on sufficiently similar variables, including rates of non-suicidal self-harm behaviour, recurrent thoughts of death, recurrent thoughts of suicide and self-injurious behaviour with any suicidal intent.

#### Substance use

Across all years of the survey, participants were asked about their lifetime and recent history of drug and alcohol use. In this study, we examined the substances that were queried across all surveys, including cigarette smoking (no data about current smoking from 2015), alcohol, cannabis, heroin, cocaine, amphetamines, methamphetamines (no data from 2003) and steroids. Specifically, we examined whether the participants reported ever using the substance and whether they had used the substance regularly (i.e. more than once per week) within the past 12 months.

#### Childhood trauma

We examined responses on the Childhood Trauma Questionnaire (CTQ; Bernstein et al., 1994), which was administered across all three years of the survey. This 28-item self-report inventory identifies five categories of maltreatment: emotional, physical and sexual abuse and emotional and physical neglect. We reported proportions of participants with moderate to extreme levels of maltreatment and also examined proportions of low to extreme abuse of any type.

### Ethics approval

Each year of the survey was approved by relevant ethics and governance bodies, including the Research Applications Subcommittee of the Department of Juvenile Justice Collaborative Research Unit (2003), the Corrections Health Service Human Research Ethics Committee (2003), the Corrective Services NSW Ethics Committee (2009), the Justice Health Human Research Ethics Committee (2009, 2015), the Juvenile Justice Research and Evaluation Steering Committee (2009, 2015) and the Aboriginal Health and Medical Research Council Human Research Ethics Committee (2003, 2009, 2015).

### Statistical analysis

IBM SPSS Statistics 27 was used for all analyses. Differences between survey years for sociodemographic and criminal justice variables were examined using chi-square analyses. Where statistically significant differences in proportions across the surveys were detected, posthoc 2 × 2 chi-square tests were conducted to compare each survey year with each other. The prevalences of mental health disorders, self-harm and suicidal behaviour, substance use and experiences of childhood trauma were assessed across comparable years using binary logistic regression, with odds ratios reported using the 2003 sample as the reference group to examine change over time. If statistically significant, we then conducted multivariate regressions adjusting for the demographic factors that changed significantly across the surveys. These adjusted odds ratios only differed from the unadjusted findings regarding one examined variable (any of the above disorders); thus, apart from this variable, our results and discussion primarily focus on unadjusted results, though both unadjusted and adjusted findings are presented in the tables.

## Results

### Sociodemographic and criminal justice characteristics

Table 1 presents a summary of the sociodemographic and criminal justice characteristics across the three survey years, with six variables found to differ significantly over time. Posthoc 2 × 2 chi-square analyses showed that the proportion of young people in custody of Aboriginal and/or Torres Strait Islander background and the proportion of those who spoke mainly English at home increased significantly between 2003 (41.7% and 80.2%) and 2015 (55.1% and 89.4%), χ^2^(1, *N* = 469) = 8.34, *p* = .004. and χ^2^(1, *N* = 468) = 7.62, *p* = .006, respectively. Conversely, the proportion of participants entering custody for the first time decreased between 2003 (27.7%) and 2015 (16.7%), χ^2^(1, *N* = 469) = 8.08, *p* = .004. The proportion of survey participants who reported not going to school in the 6 months prior to custody differed significantly between both 2003 (81.5%) and 2009 (62.1%), χ^2^(1, *N* = 538) = 23.73, *p* < .001, 2003 (81.5%) and 2015 (73.0%), χ^2^(1, *N* = 453) = 4.65, *p* = .031, and 2009 (62.1%) and 2015 (73.0%), χ^2^(1, *N* = 537) = 7.06, *p* = .008. The proportion of those with a parent who had ever been in prison increased over time, with significant differences between 2003 (42.9%) and 2015 (53.6%), χ^2^(1, *N* = 438) = 8.19, *p* = .004, and 2009 (44.6%) and 2015 (53.6%), χ^2^(1, *N* = 511) = 5.08, *p* = .024. Similarly, the proportion of participants who committed a violent offence increased between 2003 (62.4%) and 2009 (75.8%), χ^2^(1, *N* = 569) = 12.00, *p* = .001, and between 2003 (62.4%) and 2015 (74.4%;), χ^2^(1, *N* = 469) = 7.85, *p* = .005.

**Table 1.** Sociodemographic and criminal justice characteristics of participants across the 2003, 2009 and 2015 YPICHS samples.

	Sociodemographic characteristics					
	Total *N* = 800	2003 *N* = 242	2009 *N* = 331	2015 *N* = 227	χ^2^ (*df*)	*p*
	*n* (%)	*n* (%)	*n* (%)	*n* (%)		
Gender						
Male	720 (90.1)	223 (92.1)	290 (87.6)	207 (91.6)	4.00 (2)	.14
Female	79 (9.9)^a^	19 (7.9)	41 (12.4)	19 (8.4)^n^		
Aboriginal or Torres Strait Islander status	386 (48.3)	101 (41.7)	160 (48.3)	125 (55.1)	**8.34 (2)**	**.02**
Age group						
13–15 years	182 (22.8)	44 (18.2)	86 (26.0)	52 (22.9)	4.84 (2)	.09
16–21 years	618 (77.3)	198 (81.8)	245 (74.0)	175 (77.1)		
Born outside Australia	91 (11.7)^b^	36 (14.9)	36 (11.5)^j^	19 (8.4)	4.82 (2)	.09
English mainly spoken at home	663 (85.0)^c^	194 (80.2)	267 (85.6)^j^	202 (89.4)^n^	**7.92 (2)**	**.02**
Not going to school prior to custody (6 months)	543 (71.1)^d^	185 (81.5)^g^	193 (62.1)^k^	165 (73.0)^n^	**24.71 (2)**	**<** **.001**
Parent(s) ever in prison	356 (48.3)^e^	97 (42.9)^h^	139 (44.6)^l^	120 (53.6)^o^	**8.87 (2)**	**.01**
Ever placed in care (prior to age 16)	197 (25.8)^d^	64 (28.4)^i^	85 (27.2)^j^	48 (21.1)	3.73 (2)	.16
Criminal justice characteristics						
Legal status					1.46 (1)	.23
Remand		Not reported	182 (55.0)	113 (49.8)		
Sentenced		Not reported	149 (45.0)	114 (50.2)		
First time in custody	586 (73.3)^c^	67 (27.7)	75 (22.7)	38 (16.7)	**8.06 (2)**	**.02**
Violent offence	568 (71.4)^f^	151 (62.4)	248 (75.8)^m^	169 (74.4)	**13.79 (2)**	**<.01**

Note: YPICHS = Young People in Custody Health Survey.

^a^Data from 799 participants.^b^Data from 781 participants.^c^Data from 780 participants.^d^Data from 764 participants.^e^Data from 737 participants.^f^Data from 796 participants.^g^Data from 227 participants.^h^Data from 226 participants.^i^Data from 225 participants.^j^Data from 312 participants.^k^Data from 311 participants.^l^Data from 299 participants.^m^Data from 327 participants.^n^Data from 226 participants.^o^Data from 224 participants.

**Bolded** = *p* significant at the < .05 level.

### Mental health disorders

**Figure 2.**
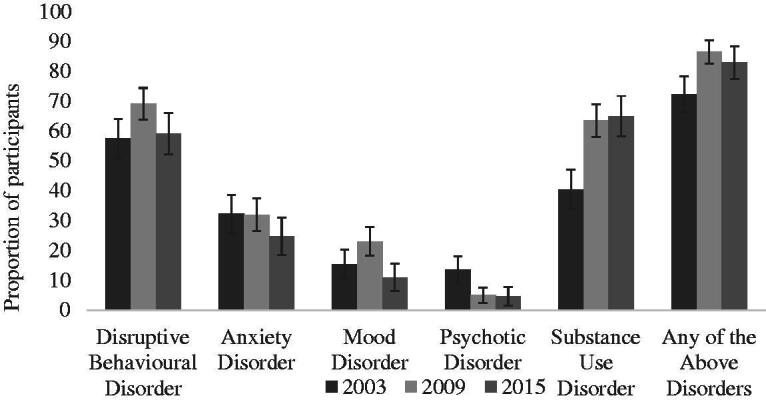
Proportions of participants diagnosed with clinical disorders in 2003, 2009 and 2015. *Note*. Adolescent Psychopathology Scale (APS) used in 2003; Kiddie Schedule for Affective Disorders and Schizophrenia for School-Age Children–Present and Lifetime version (K–SADS–PL) used in 2009 and 2015.

Table 2 presents a comparison of clinical diagnoses across the survey years, with a supporting visualisation shown in Figure 2. Strikingly, 81.3% of all participants across all three surveys had a mental disorder in at least one of the five diagnostic categories. The most prevalent disorder types across all surveys were disruptive behavioural disorders (62.9%) and substance use disorders (56.9%). Anxiety disorders (30.2%), mood disorders (17.6%) and psychotic disorders (7.6%) were less common though not infrequent.

**Table 2.** Prevalence of mental health diagnoses across the 2003, 2009 and 2015 YPICHS samples.

	Survey	*n*	Prevalence (%)	*OR*[95% CI]	*p*	a*OR* [95% CI]	*p*
Disruptive behavioural disorder	2003	217	125 (57.6)	1.00 [ref]			
	2009	293	203 (69.3)	**1.66[1.15**, **2.39]**	**<** **.01**	**1.58[1.05**, **2.37]**	.03
	2015	189	112 (59.3)	1.07 [0.72, 1.59]	.74		
Anxiety disorder	2003	213	69 (32.4)	1.00 ref			
	2009	293	94 (32.1)	0.99 [0.68, 1.44]	.94		
	2015	189	47 (24.9)	0.69 [0.45, 1.07]	.10		
Mood disorder	2003	212	33 (15.6)	1.00 [ref]			
	2009	293	68 (23.2)	**1.64[1.04**, **2.60]**	.04	**1.67[1.01**, **2.76]**	.04
	2015	189	21 (11.1)	0.68 [0.38, 1.22]	.19		
Psychotic disorder	2003	214	29 (13.6)	1.00 [ref]			
	2009	293	15 (5.1)	**0.34[0.18**, **0.66]**	**<** **.01**	**0.32[0.15**, **0.67]**	**<** **.001**
	2015	189	9 (4.8)	**0.32[0.15**, **0.69]**	**<** **.01**	**0.35[0.16**, **0.78]**	.01
Substance use disorder	2003	212	86 (40.6)	1.00 [ref]			
	2009	293	186 (63.5)	**2.57[1.79**, **3.70]**	**<** **.001**	**2.28[1.54**, **3.39]**	<.001
	2015	189	123 (65.1)	**2.73[1.82**, **4.10]**	**<** **.001**	**2.40[1.55**, **3.70]**	<.001
Any of the above disorders	2003	217	157 (72.4)	1.00 [ref]			
	2009	293	254 (86.7)	**2.49[1.59**, **3.90]**	**<** **.001**	**2.29[1.41**, **3.72]**	**<** **.01**
	2015	189	157 (83.1)	**1.88[1.16**, **3.04]**	.01	1.57 [0.94, 2.65]	.09

Note: YPICHS = Young People in Custody Health Survey; *OR* = odds ratio; a*OR =* adjusted odds ratio; CI = confidence interval. Adolescent Psychopathology Scale (APS) used in 2003;Kiddie Schedule for Affective Disorders and Schizophrenia for School-Age Children–Present and Lifetime version (K–SADS–PL) used in 2009 and 2015. Multivariate analyses include the following additional covariates: Indigenous status, language spoken at home, school attendance prior to incarceration, parental incarceration, previous incarceration and offence type.

**Bolded** = *p* significant at the < .05 level.

The prevalence of all diagnostic groups differed significantly between surveys, with the exception of anxiety disorders. There was, however, no consistent pattern of increase or decrease in prevalence over time; disruptive behavioural disorders increased significantly from 2003 (57.6%) to 2009 (69.3%), but then reduced again to a non-significant level in 2015 (59.3%). The same pattern was found for mood disorders, which increased from 15.6% to 23.2% but then dropped to 11.1%. Significant changes were also found between 2003 and the other two survey years for psychotic and substance use disorders. A greater proportion of participants had psychotic disorders in 2003 (13.6%) than in the subsequent survey years (5.1% and 4.8%). In contrast, participants had over twice the odds of having a substance use disorder in 2009 (63.5%) or 2015 (65.1%) than in 2003 (40.6%). Similarly, when examining any of the above disorders, more participants had a disorder in 2009 (86.7%) and 2015 (83.1%) than in 2003 (72.4%). However, after adjusting for the significant demographic factors, this increase remained significant only between 2003 and 2009, as the rate declined slightly in 2015.

### Self-harm and suicidal behaviours

A comparison of self-harm and suicide variables from the 2003 and 2009 surveys is presented in Table 3. Of all participants in the 2003 and 2009 surveys, 19.7% reported lifetime self-harm ideation, and 14.6% reported actual self-harm in their lifetime, with 10.1% reporting self-harm within the past 12 months. In terms of suicide, 17.2% reported lifetime ideation, 11.6% reported suicidal ideation within the past 12 months, and 9.1% reported actually attempting suicide within the past 12 months. No statistically significant differences were found between 2003 and 2009, with proportions remaining largely stable across the two surveys.

**Table 3.** Prevalence of self-reported self-harm and suicidal behaviours across the 2003 and 2009 YPICHS samples.

	Survey	Prevalence (%)	*OR* [95% CI]	*p*
Lifetime self-harm ideation	2003	40 (18.2)	1.00 [ref]	
	2009	65 (20.7)	1.18 [0.76, 1.82]	.47
Self-harm ideation within past 12 months	2003	27 (12.3)	1.00 [ref]	
	2009	36 (11.5)	0.93 [0.54, 1.58]	.78
Lifetime self-harm	2003	27 (12.3)	1.00 [ref]	
	2009	51 (16.2)	1.39 [0.84, 2.29]	.20
Self-harm within past 12 months	2003	20 (9.4)	1.00 [ref]	
	2009	34 (10.8)	1.21 [0.68, 2.17]	.51
Lifetime suicidal ideation	2003	42 (19.1)	1.00 [ref]	
	2009	50 (15.9)	0.80 [0.51, 1.26]	.34
Suicidal ideation within past 12 months	2003	32 (14.5)	1.00 [ref]	
	2009	30 (9.6)	0.62 [0.37, 1.06]	.08
Suicide attempt within past 12 months	2003^a^	18 (8.4)	1.00 [ref]	
	2009	30 (9.6)	1.16 [0.63, 2.13]	.64

Note: YPICHS = Young People in Custody Health Survey; *OR* = odds ratio; CI = confidence interval. Data from 220 participants in 2003 and 314 participants in 2009.

^a^Data from 215 participants.

Participants in 2015 were not asked these specific self-harm and suicidal behaviour questions, and thus a direct comparison was not possible. However, a different screening measure focusing on self-harm and suicidal behaviour was included in the K–SADS screening that year. Over the 12 months before the interview, 5.5% of participants engaged in repetitive non-suicidal self-harm behaviour (i.e. more than five times and/or with significant injury to the self). In terms of suicidal behaviour, 10.2% reported recurrent thoughts of death (i.e. ‘I would be better off dead’ or ‘I wish I were dead’), and 3.3% reported recurrent thoughts of suicide. Over the past year, 9.7% of participants reported any self-injurious behaviour with any suicidal intent, a similar rate to that reported by participants in 2003 (8.4%) and 2009 (9.6%).

### Substance use

**Figure 3.**
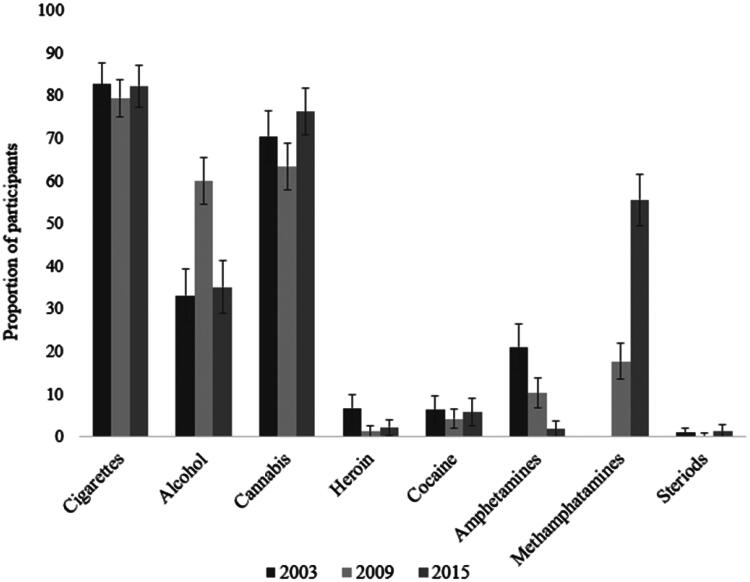
Proportions of participants who regularly used substances within the 12 months prior to custody. *Note.* Regular use: used at least once per week.

High levels of regular drug and alcohol use were reported across all three years of the survey, as shown in Table 4 and visualised in Figure 3.^1^ For example, 81.3% of all participants reported smoking cigarettes more than one day per week within the past 12 months, and 44.9% of participants reported being drunk more than once per week in the past 12 months. Over two-thirds of participants across survey years also reported using cannabis at least once per week.

**Table 4.** Prevalence of regular substance use across the 2003, 2009 and 2015 YPICHS samples.

	Year (*n*)	Prevalence (%)	OR [95% CI]	*p*	a*OR* [95% CI]	*p*
Smoked cigarettes (past 12 months)	2003 (225)	186 (82.7)	1.00 [ref]			
	2009 (317)	252 (79.5)	0.81 [0.52, 1.26]	.36		
	2015 (226)	186 (82.3)	0.98 [0.60, 1.59]	.92		
Smoke cigarettes (currently)	2003 (217)	127 (58.0)	1.00 [ref]			
	2009 (317)	67 (21.1)	**0.19[0.13**, **0.28]**	**<** **.001**	**0.19[0.12**, **0.28]**	**<** **.001**
Drunk	2003 (217)	72 (33.2)	1.00 [ref]			
	2009 (315)	189 (60.0)	**3.02[2.10**, **4.34]**	**<** **.001**	**2.68[1.83**, **3.92]**	**<** **.001**
	2015 (225)	79 (35.1)	1.09 [0.74, 1.62]	.67		
Cannabis	2003 (224)	158 (70.5)	1.00 [ref]			
	2009 (299)	190 (63.5)	0.73 [0.50, 1.06]	.09		
	2015 (224)	171 (76.3)	1.35 [0.88, 2.05]	.17		
Heroin	2003 (223)	22 (9.9)	1.00 [ref]			
	2009 (317)	4 (1.3)	**0.12[0.04**, **0.34]**	**<** **.001**	**0.14[0.05**, **0.42]**	**<** **.001**
	2015 (225)	5 (2.2)	**0.21[0.08**, **0.56]**	**<** **.01**	**0.18[0.07**, **0.51]**	**<** **.01**
Cocaine	2003 (224)	14 (6.3)	1.00 [ref]			
	2009 (313)	13 (4.2)	0.64 [0.30, 1.40]	.27		
	2015 (225)	13 (5.8)	0.91 [0.42, 1.99]	.82		
Amphetamines	2003 (223)	47 (21.1)	1.00 [ref]			
	2009 (312)	32 (10.3)	**0.43[0.26**, **0.70]**	**<** **.01**	**0.41[0.24**, **0.69]**	**<** **.01**
	2015 (225)	4 (1.8)	**0.07[0.02**, **0.19]**	**<** **.001**	**0.05[0.02**, **0.16]**	**<** **.001**
Methamphetamines	2003	–				
	2009 (314)	16 (5.1)	1.00 [ref]			
	2015 (225)	67 (29.8)	**7.90[4.43**, **14.08]**	**<** **.001**	**6.74[2.67**, **12.35]**	**<** **.001**
Steroids	2003 (224)	2 (0.9)	1.00 [ref]			
	2009(316)	1 (0.3)	0.35 [0.03, 3.91]	.40		
	2015 (225)	3 (1.3)	1.50 [0.25, 9.06]	.66		

Note: YPICHS = Young People in Custody Health Survey; *OR* = odds ratio; a*OR =* adjusted odds ratio; CI = confidence interval. Regular use: used at least once per week (within past 12 months). Multivariate analyses include the following additional covariates: Indigenous status, language spoken at home, school attendance prior to incarceration, parental incarceration, previous incarceration and offence type.

**Bolded** = *p* significant at the < .05 level.

The use of some substances declined over time, whereas the use of others increased. Reports of current cigarette smoking (only available in 2003 and 2009) drastically decreased over time, from 58.0% reporting smoking at least one day per week in 2003 to only 21.1% of participants in 2009. The proportion of participants who regularly used heroin also dropped significantly over the years (9.9% to 1.3% to 2.2%). So did the proportion of participants who had ever tried heroin (odds ratio, *OR* = 0.26,95% confidence interval, CI [0.15, 0.46], *p* < .001; *OR* = 0.40,95% CI [0.23, 0.70], *p* = .001): nearly 20% of participants reported having tried heroin in 2003, whereas only 6.0% and 8.9% had tried heroin in 2009 and 2015, respectively. A similar pattern was seen for amphetamine use (excluding methamphetamine): 47.3% of participants had tried amphetamines in 2003, 29.3% in 2009 and 18.7% in 2015 (*OR* = 0.46,95% CI [0.23, 0.66], *p* < .001; *OR* = 0.23,95% CI [0.17, 0.39], *p* < .001). On the other hand, methamphetamine use (only available for 2009 and 2015) significantly increased over time, in regard to both regular use and methamphetamine use ever (*OR* = 5.83,95% CI [3.94, 8.61], *p* < .001). In 2009, 17.7% of participants had used methamphetamine, and 5.1% used it regularly; by 2015, over half of participants had used the drug, with nearly a third using it at least once per week. Additionally, in 2009, participants were three times more likely to report being regularly drunk than in 2003 (60.0% vs. 33.2%), though this number fell again in 2015 (35.1%). The proportion of participants who had ever used cocaine increased from 2003 (21.0%) and 2009 (20.2%) to 2015 (31.6%; *OR* = 1.74,95% CI [1.13, 2.66], *p* = .011), though rates of regular cocaine use did not change significantly over time.

### Childhood trauma

A summary of the proportion of survey samples reporting traumatic childhood experiences is presented in Table 5. In general, high rates of childhood trauma were observed, with 64.5% of participants across all years experiencing any level of abuse. The most common type of moderate to extreme maltreatment reported was physical abuse, which was experienced by 25.8% of all participants. Even sexual abuse, the least commonly reported type of moderate to extreme maltreatment, was still experienced by 7.5% of participants over the three surveys.

**Table 5.** revalence of self-reported childhood trauma across the 2003, 2009 and 2015 YPICHS samples.

	Year (*n*)	Prevalence (%)	*OR* [95% CI]	*p*	a*OR* [95% CI]	*p*
Moderate to extreme emotional abuse	2003 (218)	33 (15.1)	1.00 [ref]			
	2009 (307)	48 (15.6)	1.04 [0.64, 1.68]	.88		
	2015 (189)	36 (19.0)	1.32 [0.79, 2.22]	.30		
Moderate to extreme physical abuse	2003 (220)	65 (29.5)	1.00 [ref]			
	2009 (307)	71 (23.1)	0.72 [0.48, 1.06]	.10		
	2015 (189)	49 (25.9)	0.84 [0.54, 1.29]	.42		
Moderate to extreme sexual abuse	2003 (220)	15 (6.8)	1.00 [ref]			
	2009 (307)	23 (7.5)	1.11 [0.56, 2.17]	.77		
	2015 (189)	16 (8.5)	1.26 [0.61, 2.63]	.53		
Moderate to extreme emotional neglect	2003 (219)	34 (15.5)	1.00 [ref]			
	2009 (307)	51 (16.6)	1.08 [0.68, 1.74]	.74		
	2015 (189)	38 (20.1)	1.37 [0.82, 2.28]	.23		
Moderate to extreme physical neglect	2003 (219)	37 (16.9)	1.00 [ref]			
	2009 (307)	69 (22.5)	1.43 [0.92, 2.22]	.12		
	2015 (189)	52 (27.5)	**1.87[1.16**, **3.01]**	.01	**1.91[1.14**, **3.18]**	.01
Abuse of any type (low, moderate, severe or extreme)	2003 (216)	147 (68.1)	1.00 [ref]			
	2009 (307)	184 (59.9)	0.70 [0.49, 1.01]	.06		
	2015 (189)	128 (67.7)	0.99 [0.65, 1.50]	.94		

Note: YPICHS = Young People in Custody Health Survey; *OR* = odds ratio; a*OR =* adjusted odds ratio; CI = confidence interval. Scores from the Childhood Trauma Questionnaire. Multivariate analyses include the following additional covariates: Indigenous status, language spoken at home, school attendance prior to incarceration, parental incarceration, previous incarceration and offence type.

**Bolded** = *p* significant at the < .05 level.

Proportions of participants who experienced childhood trauma did not differ much across the surveys. The only statistically significant difference found was for physical neglect, with participants in 2015 being almost twice as likely to report an experience of physical neglect than participants in 2003.

## Discussion

In this study comparing three surveys of young people in custody conducted in 2003, 2009 and 2015 in NSW, the prevalence of mental illnesses was found to differ over time, but no consistent pattern was observed. However, in each survey the majority of young people were found to have at least one mental illness, with the proportion increasing from around 72.4% in 2003 to 86.7% in 2009 and then dropping slightly to 83.1% in 2015. While the overall stability of mental illness prevalence amongst young people in custody was similar to that seen in the general population, the trend towards decreasing rates of cigarette smoking and excessive alcohol use seen amongst young people in the general population did not appear to extend to those young people detained in custody. These ongoing high rates of mental illness and substance use highlight areas where further interventions or treatments are essential.

### Mental health disorders

With regard to individual mental illness diagnoses, variable patterns of prevalence were seen over the three surveys. Disruptive behavioural disorders and mood disorders increased in prevalence between 2003 and 2009, and then fell again in 2015, while the proportion of young people diagnosed with psychotic disorders decreased between 2003 and the subsequent survey years. Overall, the proportion of substance use disorders diagnosed increased, while the prevalence of anxiety disorders remained stable. It is important to note that the APS instrument was used to diagnose mental illness in 2003 whereas the more formal and widely used diagnostic tool, the K–SADS–PL, was used in 2009 and 2015. This change in clinical tool may have contributed to some of the differences in disorder rates seen. In sum, enduring increases or decreases in the prevalence of mental illness diagnoses were not observed but rates were very high across the spectrum and over time. These results align with a recent large meta-analysis that found stable estimates of the prevalence of mental illness in adolescents in juvenile detention across studies conducted over time (Beaudry et al., 2021).

Rates of mental illness in young people in the Australian general population are regularly determined through repeat surveys. These existing surveys of Australian children and adolescents largely also show stability in the prevalence of mental disorders across many types of mental disorders over time. For example, the Young Minds Matter surveys conducted in 1998 and 2013–2014 showed little change in proportions of those aged 12–17 diagnosed with ADHD (7–6%) and conduct disorder (2% both years), with major depressive disorder increasing slightly from 3% to 5% across those years (Lawrence et al., 2015, 2016). Similarly, disruptive behavioural disorders and mood disorders in the current study’s sample of young people in custody did not significantly change between 2003 and 2015. However, the relative difference in prevalence rate between custody and general community samples remains striking, since 62.9% of young people across YPICHS had a disruptive behavioural disorder, and 17.6% had a mood disorder.

Another adolescent survey conducted in 2007 by the Australian Bureau of Statistics examined three disorder types in a slightly older sample of young people. Over a quarter of those aged 16–24 years had at least one mental disorder (26%): 15% had an anxiety disorder, 6% had an affective disorder, and 13% had a substance use disorder (Australian Bureau of Statistics, ABS, 2007). When compared with the closest YPICHS (i.e. 2009), much larger prevalences for young people in custody were again apparent.

### Self-harm and suicidal behaviours

We found little change over time in regard to self-harm and suicidal behaviours amongst young people in custody, with such behaviours being reported by a significant minority in each survey. For example, between 8.4% and 9.7% of participants across all three surveys reported self-harm behaviour with suicidal intent in the past year. This rate was much higher than that found in the 1998 and 2013–2014 Young Minds Matter Surveys, where 4% and 2%, respectively, of those aged 12–17 years reported attempting suicide within the previous 12 months (Sawyer et al., 2001; Zubrick et al., 2016). Similarly, 10.1% of young people in custody in 2003 and 2009 reported self-harm, and 11.6% reported suicidal ideation in the past 12 months, compared with 8% of general population adolescents in 2013–2014 for both these variables. Thus, as with mental disorders, we see largely stable but elevated rates of self-harm and suicidal behaviours in young people in custody compared to those in the general population.

### Substance use

In the current study, some changes in rates of reported alcohol and drug use were observed over time amongst young people in custody. Specifically, decreases in the reported rates of current cigarette smoking, lifetime and regular heroin use and lifetime and regular amphetamine use were seen. On the other hand, there were sustained increases in lifetime cocaine use and lifetime and regular methamphetamine use.

The National Drug Strategy Household Surveys provide insight into trends over time in the general Australian population with regard to alcohol and drug use (Australian Institute of Health and Welfare, AIHW, 2001, 2010, 2016). Cigarette smoking steadily decreased over time in those aged 18–24 years in the general population, with 24% daily and 8% occasional smokers in 2001, 16% daily and 5% occasional smokers in 2010 and 12% daily and 4% occasional smokers in 2016. Additionally, 10% of 13–17-year-old participants in the 2013–2014 Young Minds Matter survey reported smoking at least once a week at some point in their lives (Lawrence et al., 2015). Though rates of current smokers in the YPICHS samples decreased between 2003 and 2009, around 80% of the total sample in each survey reported regular cigarette smoking (i.e. at least once per week within past 12 months). Therefore, although rates of cigarette smoking decreased in the general population, the same was not true for young people in custody, highlighting an ongoing problem for this population.

Rates of excessive alcohol use in young people in the general population in Australia have also decreased over time. Over half of 18–24-year-olds and 21% of 14–17-year-olds reported drinking more than four drinks at a time per month in 2010, but by 2016 these numbers had dropped to 42% and 8%, respectively (AIHW, 2010, 2016). Rates for young people in custody were higher but fluctuated over the three surveys, with 33.2% reporting being drunk more than once per week during the past 12 months in 2003, 60.0% in 2009 and 35.1% in 2015.

Finally, rates of other drug use were higher in the sample of young people in custody than in the general population. For example, regular use of cannabis (i.e. more than once per week) amongst YPICHS participants was 70.5%, 63.5% and 76.3% over the three surveys. In comparison, 32% (2001), 22% (2010) and 25% (2016) of 18–24-year-olds in the general population had recently used cannabis (i.e. used at some point within the past 12 months), with even lower rates for younger adolescents (aged 14–17 years; AIHW, 2001, 2010, 2016). Only 5% of younger adolescents (13–17 years) reported using cannabis within the past month in a 2013–2014 survey (Lawrence et al., 2015). Rates of cocaine and meth/amphetamine use were similarly lower in general population adolescents, even with this less stringent definition (i.e.recent use vs. regular use; AIHW, 2001, 2010, 2016). Though trends over time varied by substance, these comparative data generally show the ongoing heightened substance use rates for young people in custody. The high rates of substance use clearly represent an ongoing problem, particularly given that – in addition – over half of the participants across the YPICHS were diagnosed with a substance use disorder.

### Childhood trauma

We found that experiences of childhood trauma were common in the samples of young people in custody, with 64.5% of the total sample reporting having experienced at least one type of maltreatment. No changes over time were observed apart from an increase in reported physical neglect, which rose from 16.9% in 2003 to 27.5% in 2015. Limited evidence exists to establish the prevalence of childhood maltreatment in Australia (see Child Family Community Australia, CFCA, 2017; Mathews et al., 2016) with no relevant data tracking changes over time. Still, in a relevant meta-analysis, Moore et al. (2015) collated data from studies across Australia and reported the pooled prevalence rates for different types of abuse. Experiences of abuse were based on retrospective self-report by the victims in all studies, and the definition of childhood in each study ranged from under 15 to under 18 years of age. They found that around 9% of children experienced physical abuse, 9% experienced emotional abuse, 9% experienced sexual abuse, and around 2% experienced neglect (including physical or emotional neglect). Additionally, the 2016 Personal Safety Survey asked adults in Australia about their experiences of childhood abuse (abuse before the age of 15) and found similar rates, with 9% reporting childhood physical abuse and 8% reporting childhood sexual abuse (ABS, 2016). Participants in YPICHS reported greater levels of moderate to extreme emotional abuse, physical abuse, emotional neglect and physical neglect. Between 6.8% and 8.5% of YPICHS participants reported moderate to severe sexual abuse depending on the survey year, in line with national levels, although a high threshold of moderate to severe abuse was included in the definition for young people in custody. Overall, these data suggest that young people in custody experience substantially higher rates of childhood abuse or neglect than young people in the general population. The increasing rate of self-reported physical neglect is of concern but may also reflect changes over time in the young people’s understanding and willingness to disclose abuse, including when experienced in the form of neglect.

### Strengths and limitations

This study is one of the first in Australia to examine key variables – including the prevalence of mental illness, self-harm and suicidal behaviours, substance use and childhood abuse – over time within young people in custody, a population in a setting that is difficult to access. The three surveys used similar sampling and methodology and were conducted in the same setting, allowing for robust examinations of changes over time. Another strength of this study is the use of a structured diagnostic tool (i.e.K–SADS–PL) in 2009 and 2015 for mental illness diagnoses, helping increase validity and ensuring more reliable results (Kaufman et al., 1997). There was a strong response rate among the young people eligible for participation with 75% to 90% of invited young people consenting across survey years. Finally, we were also able to make informative comparisons using general population data regarding the prevalences and trends of related variables.

The current study was not without limitations. Certain methodological variations in the surveys restricted some comparisons, such as the different self-harm and suicide questions that were asked in the 2015 survey. Similarly, the APS tool was used to establish mental illness diagnoses in 2003 and the K–SADS–PL used in 2009 and 2015. Thus, our comparison of mental illness across the surveys comes with a caveat, particularly given that the APS is not a formal diagnostic tool. As such, the APS may over- or underestimate mental illness diagnoses. For example, some research suggests that tools that score highly for psychotic-like experiences may overestimate psychotic disorders (e.g. Schultze-Lutter et al., 2014), possibly explaining the much higher rate of psychotic disorders in our sample in 2003 (13.6%) than in later years (5.1% and 4.8%). Other questions, such as those regarding drug and alcohol use and childhood trauma, remained unchanged across surveys.

The exclusion criteria applied may affect the generalisability of the findings, including with regard to underestimating the prevalence rates for serious mental illness or substance use problems given that individuals lacking capacity to consent to participate may have been acutely mentally unwell, suffering acute substance withdrawal, or considered too violent or disruptive by custody centre staff. While the survey sample sizes are large relative to previous studies, some comparisons may have been limited by inadequate power to detect differences present over time.

Finally, further changes in the prevalence of mental health problems amongst young people in custody may have occurred since the last survey conducted in 2015. Indeed, research has already shown the impact of the COVID-19 pandemic on young people’s mental health and self-harm or suicidal behaviours in both custodial (e.g. Johnson, Gutridge, Parkes, Roy, & Plugge, 2021; Kasinathan et al., 2021) and community (e.g. Corrigan et al., 2022; Jones, Mitra, & Bhuiyan, 2021; Sara et al., 2022; Zolopa et al., 2022) settings. Additionally, recent data suggest increases in mental health problems in young people in the general population in recent years (ABS, 2022), and these increases could also be reflected in custody. Ongoing tracking of mental health prevalence over time amongst young people in custody is important and would also allow for the identification and investigation of key changes over time, helping to inform custodial adolescent mental health and drug and alcohol services and interventions aimed at diverting young people with mental health problems away from the criminal justice system.

## Implications and conclusions

These data, collected over a 12-year period, provided insight into the ongoing mental health concerns of young people in custody. Though we observed some fluctuation over time, overall rates of mental illness generally remained stable in this population, largely consistent with contemporaneous patterns in the general population. However, the persistence of high rates of mental illness amongst young people in custody in NSW indicates a failure of early community intervention and diversionary interventions aimed at reducing the risk of young people with mental health problems entering the criminal justice system, as well as remaining in the system as adults. Concerningly, the prevalence rates for mental illness and self-harm and suicidal behaviours were substantially greater in young people in custody than in those in the general population, a discrepancy that has also persisted over time. Substance use rates amongst young people in custody were also remarkably high, with persistently high levels of regular nicotine, alcohol and cannabis use, at odds with reductions in prevalence seen amongst young people in the general population over the same period. Although reduced rates of self-reported use of some substances were found (e.g. heroin use, current cigarette smoking), rates for other substances grew considerably, most notably methamphetamine. Finally, rates of childhood trauma were greater than those seen in the general population, with rates either remaining the same or increasing over time. In sum, these data suggest little improvement in the well-being of young people in custody in New South Wales over time. Better identification and treatment of mental health problems, self-harm and suicidal behaviours and alcohol and drug problems are vital if young people are to be prevented from becoming enmeshed in the criminal justice system.

## Supplementary Material

Supplemental Material

## Footnotes

1 See Table A in Supplementary Materials for the prevalence of substances *ever* used.
