# Supplementary material for: The prevalence of mental illness in young people in custody over time: a comparison of three surveys in New South Wales
Source: Psychiatr Psychol Law. 2023 May 31;31(2):235–53. doi: 10.1080/13218719.2023.2192257 (PMC11018085; doi:10.1080/13218719.2023.2192257)
Supplement: Supplemental Material [file TPPL_A_2192257_SM2361.docx]

**Table A**

Prevalence of Substance Ever Used across the 2003, 2009, and 2015 YPICHS Samples

|  |  | **Prevalence** | **OR (95% CI)** | ***p*-value** | **aOR (95% CI)** | ***p*-value** |
| --- | --- | --- | --- | --- | --- | --- |
| Smoked a cigarette | 2003 (*n* = 226) | 213 (94.2%) | 1.00 (ref) |  |  |  |
|  | 2009 (*n* = 317) | 298 (94.0%) | 0.96 (0.46-1.98) | .906 | 0.87 (0.41-1.85) | .709 |
|  | 2015 (*n* = 226) | 208 (92.0%) | 0.71 (0.34-1.48) | .354 | 0.61 (0.28-1.33) | .215 |
| Had a full serving of alcohol | 2003 (*n* = 226) | 212 (93.8%) | 1.00 (ref) |  |  |  |
|  | 2009 (*n* = 317) | 300 (94.6%) | 1.17 (0.56-2.42) | .681 | 1.03 (0.47-2.26) | .940 |
|  | 2015 (*n* = 226) | 211 (93.4%) | 0.93 (0.44-1.97) | .929 | 0.73 (0.33-1.59) | .427 |
| Been drunk | 2003 (*n* = 226) | 193 (85.4%) | 1.00 (ref) |  |  |  |
|  | 2009 (*n* = 317) | 287 (90.5%) | 1.64 (0.97-2.77) | .067 | 1.50 (0.85-2.63) | .160 |
|  | 2015 (*n* = 226) | 204 (90.3%) | 1.59 (0.89-2.82) | .116 | 1.27 (0.70-2.31) | .432 |
| Cannabis | 2003 (*n* = 224) | 198 (88.4%) | 1.00 (ref) |  |  |  |
|  | 2009 (*n* = 317) | 276 (87.1%) | 0.88 (0.52-1.49) | .645 | 0.87 (0.50-1.52) | .627 |
|  | 2015 (*n* = 225) | 203 (90.2%) | 1.21 (0.67-2.21) | .531 | 1.08 (0.57-2.03) | .816 |
| Heroin | 2003 (*n* = 224) | 44 (19.6%) | 1.00 (ref) |  |  |  |
|  | 2009 (*n* = 317) | 19 (6.0%) | **0.26 (0.15-0.46)** | **< .001** | **0.25 (0.13-0.46)** | **< .001** |
|  | 2015 (*n* = 225) | 20 (8.9%) | **0.40 (0.23-0.70)** | **.001** | **0.29 (0.15-0.55)** | **< .001** |
| Cocaine | 2003 (*n* = 224) | 47 (21.0%) | 1.00 (ref) |  |  |  |
|  | 2009 (*n* = 317) | 64 (20.2%) | 0.95 (0.62-1.45) | .822 | 0.94 (0.59-1.47) | .775 |
|  | 2015 (*n* = 225) | 71 (31.6%) | **1.74 (1.13-2.66)** | **.011** | **1.71 (1.08-2.71)** | **.021** |
| Amphetamines | 2003 (*n* = 224) | 106 (47.3%) | 1.00 (ref) |  |  |  |
|  | 2009 (*n* = 317) | 93 (29.3%) | **0.46 (0.32-0.66)** | **< .001** | **0.47 (0.32-0.69)** | **< .001** |
|  | 2015 (*n* = 225) | 42 (18.7%) | **0.23 (0.17-0.39)** | **< .001** | **0.23 (0.14-0.36)** | **< .001** |
| Methamphetamines | 2003 | - |  |  |  |  |
|  | 2009 (*n* = 317) | 56 (17.7%) | 1.00 (ref) |  |  |  |
|  | 2015 (*n* = 225) | 125 (55.6%) | **5.83 (3.94-8.61)** | **< .001** | **5.22 (3.42-7.96)** | **< .001** |
| Steroids | 2003 (*n* = 224) | 4 (1.8%) | 1.00 (ref) |  |  |  |
|  | 2009 (*n* = 316) | 4 (1.3%) | 0.71 (0.17-2.85) | .624 | 0.92 (0.22-3.84) | .913 |
|  | 2015 (*n* = 225) | 7 (3.1%) | 1.77 (0.51-6.12) | .370 | 1.74 (0.47-6.48) | .410 |

*Note.* Multivariate analyses include the following additional covariates: Indigenous status, language spoken at home, school attendance prior to incarceration, parental incarceration, previous incarceration, and offence type.
